# Shikonin induces apoptosis and autophagy via downregulation of pyrroline-5-carboxylate reductase1 in hepatocellular carcinoma cells

**DOI:** 10.1080/21655979.2022.2052673

**Published:** 2022-03-16

**Authors:** Junli Zhang, Ling Shang, Wendi Jiang, Wenjuan Wu

**Affiliations:** a Bengbu Medical College Key Laboratory of Cancer Research and Clinical Laboratory Diagnosis, Bengbu Medical College, Bengbu, China; b Department of Biochemistry and Molecular Biology, School of Laboratory Medicine, Bengbu Medical College, Bengbu, China

**Keywords:** Shikonin, PYCR1, apoptosis, autophagy, hepatocellular carcinoma

## Abstract

Shikonin(SK) is a natural small molecule naphthoquinone compound, which has anti-cancer activity in various human malignant tumors. Pyrroline-5-carboxylate reductase 1(PYCR1) is involved in tumorigenesis and regulates various cellular processes, including growth, invasion, migration, and apoptosis. However, the effect of SK and PYCR1 on apoptosis and autophagy in hepatocellular carcinoma are unclear. Our goal is to determine the internal molecular mechanism of the interaction between SK and PYCR1 and its role in the occurrence and development of liver cancer. The CCK8 assay, wound healing assay, and transwell assays show that SK and siPYCR1(gene silence PYCR1) inhibited the malignant phenotype of HCC cells, including cell viability, colony formation, migration, and invasion, respectively. The flow cytometry assays and immunofluorescence show that SK and siPYCR1 activated apoptosis and autophagy, respectively. SK induces apoptosis and autophagy in a dose-dependent manner. In addition, HCC cells were transfected with small interference fragment PYCR1 siRNA to construct siPYCR1 and SK single treatment group and co-treatment group to verify the interaction between SK and PYCR1. The Western blot identified that PI3K/Akt/mTOR signal pathway protein expression was significantly downregulated in HCC cells treated with SK and siPYCR1 together. Collectively, SK may induce apoptosis and autophagy by reducing the expression of PYCR1 and suppressing PI3K/Akt/mTOR. Thus, SK may be a promising antineoplastic drug in Hepatocellular carcinoma (HCC). SK downregulating PYCR1 might supply a theoretical foundation for the potential therapeutic application in hepatocellular carcinoma.

## Graphical abstract

**Figure:**
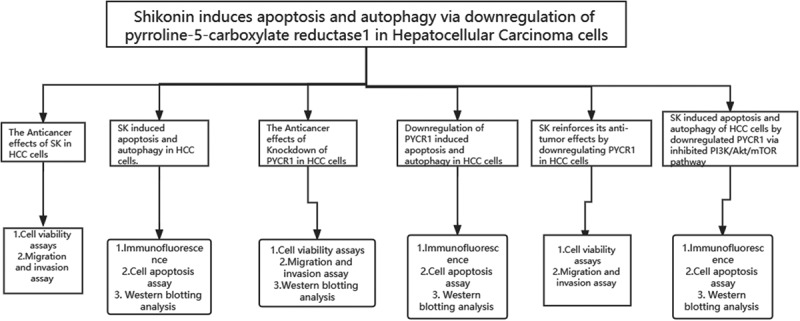

## Introduction

Liver cancer holds the third principle reason for cancer-associated death globally and remains at high levels of morbidity [1]. HCC is the common histologic subtype of liver cancer, accounting for 90% of primary liver cancer [2]. It has the characteristics of easy recurrence, uncomplicated metastasis, and lack of specific symptoms in the late stage [3]. Although, in recent years, with the continuous progress of surgical hepatic resection and liver transplantation techniques and the improvement of traditional chemotherapy, the effect of all treatment methods is still unsatisfactory [4]. Also, the molecular mechanism of hepatocarcinogenesis and chemoresistance remains ambiguous. Systemic radiotherapy and chemotherapy have many side effects [2]. Therefore, to improve the level of early diagnosis and targeted therapy and significantly improve the prognosis of patients, it is urgent to develop new liver cancer treatment strategies and find potential new treatment targets.

The proline metabolic cycle has a vast impact on the growth and survival of cancer cells [5], suggesting a variety of enzymes involved in the cycle may become the target of therapeutic intervention. PYCR1 is a principal rate-limiting enzyme that catalyzes pyrrolidine-5-carboxylic acid (P5C) to synthesize proline [6], indicating PYCR1 may be involved in the oncogenesis of some kinds of tumors. Early studies found that human PYCR1 gene mutation is associated with autosomal recessive cutis laxa, cellular oxidative stress, and mitochondrial function impairment [7–9]. In recent years, the impact of PYCR1 in many cancers cannot be ignored or underestimated. In some studies, high expression levels of PYCR1 have been recorded in multiple cancers, including HCC, breast cancer, and lung cancer, promoting the development and progression of tumors and is contrary to the overall survival rate [10–12]. For example, PYCR1 was downregulated by miR-488, which promoted cell proliferation and inhibited cell apoptosis, and activated the p38/MAPK pathway in non-small-cell lung cancer (NSCLC) [13]. In HCC, PYCR1 gene interference can restrain the malignant phenotype of HCC cells by inhibiting activation of the Akt pathway [14]. Another study discovered that the PYCR1 gene deletion suppresses cell proliferation and encourages cell apoptosis of HCC cells by the c-Jun N-terminal kinase/insulin receptor substrate1 (JNK/IRS1) pathway [10]. The detailed function and potential mechanism of PYCR1 in liver cancer are still unclear to a great extent. Thus, it is valuable to study the role of the PYCR1 gene in liver cancer.

SK is a natural active compound divided from the Chinese herbal plant *Lithospermum erythrorhizon* [15]. It has been certified to possess multiple biological activities, including enhancing immunity, antiviral, anti-inflammatory, anti-cancer, and anti-ischemia/reperfusion injury [16,17]. SK has negligible damage to normal cells and makes an excellent antineoplastic impact on many tumor cells [18,19]. For instance, SK retards the proliferation of tumor cells, such as liver cancer, breast cancer, and gastric cancer, and accelerates cell apoptosis [20–22]. Moreover, SK modulates the galectin-1/JNK signaling axis to stimulate colorectal carcinoma cells apoptosis and autophagy [23]. Nevertheless, in human melanoma A375 cells, SK induces cell apoptosis and cell autophagy via activating ROS-mediated ER stress and p38 pathways [24]. These findings suggest that SK shows the potential to treat cancer, but the function of SK on autophagy and apoptosis in hepatocellular carcinoma has not been further studied.

Thus, this study aims to explore how SK regulates autophagy and apoptosis of hepatoma cells. We used cell function tests to clarify the effect of SK and PYCR1 on the growth, migration, invasion, the link among SK between PYCR1, and focused on their role in autophagy and apoptosis of HCC cells. SK may activate autophagy and apoptosis of SNU-449 and Hep-3B cells by down-regulating PYCR1. In studying the mechanism of SK in autophagy and apoptosis of HCC cells, we also found that SK downregulated the expression of PYCR1 and then inhibited PI3K/Akt/mTOR signal pathway to activate autophagy and apoptosis. These results enable us to understand better the molecular mechanism of SK in tumor autophagy and apoptosis and facilitate the discovery of persuasive methods for treating liver cancer.

## Materials and methods

### Cell culture

The human HCC cells lines (LO2, SMMC-7721, Huh-7, Hep-3B, SNU-449, BEL-7404, and HepG2) were purchased from the Chinese Academy of Sciences (Shanghai, China). Cells were grown in DMEM (Gibco, USA) with high glucose and RPMI‑1640 medium (Gibco, USA), supplemented with heat-inactivated 10% fetal bovine serum (FBS) (Gibco, USA) and 1% Penicillin/streptomycin (Gibco, USA) in 5% CO^2^ incubator.

### Chemicals and antibodies

Shikimin, acquired from MedChemExpress (MCE, USA). SK was analyzed by HPLC and is over 98%. The antibodies used were as following: PYCR1(1:3000), PI3K(1;500), Akt(1:1000), phospho-Akt(1:1000), mTOR(1:1000), β-actin(1:5000), ULK(1:1000), phospho-ULK(1:1000), Beclin-1(1:1000), p62(1:1000), -caspase3(1:1000), PARP(1:2000), Bax(1:2000), and Bcl-2(1:1000), which came from ProteinTech Group (Chicago, USA). Antibodies for LC3-I/II(1:2000), caspase9(1:1000), phospho-mTOR(1:1000), and phospho-PI3K(1:2000) were purchased from Cell Signaling Technology (Boston, USA).

### Cell viability assays

Cell viability assays was conducted according to the method described in previous study [25]. SNU-449 and Hep-3B cells (5 × 103 per well) were seeded into a 96-well plate in a 5% CO^2^ incubator overnight. SNU-449 and Hep-3B cells were respectively exposed to various doses of SK (0, 0.25, 0.5, 1, 2, 4 and 8 µM) (0, 0.5, 1, 2, 4, 8 and 16 µM) for 24 h, 48 h, 72 h and 96 h or transfected with PYCR1 siRNA for 48 h. Then, the cell counting kit (CCK)-8 solution (10 μL) (Beyotime, Shanghai, China) was added to each well. Absorbance at 450 nm was measured using a Microplate Reader (ELx800, BioTek, USA).

### Cell apoptosis assay

According to previous test methods [26], to detect early and late apoptosis, using Annexin V-FITC/PI dual staining detection kits (Beyotime, Shanghai, China). Cells(1 × 10^6^) were stained with 5 µL of Annexin V and 10 µL of PI in 198 μL of 1× binding buffer for 15 min at room temperature in the dark. Apoptotic cells were detected using a flow cytometer (Cytomics FC500). FlowJo software calculates the apoptosis rate.

### Migration and invasion assay

For the wound healing assay, a scraped area was artificially created using a 10 μL pipette tip. When the cell density reached about 90%, the well plates were added with serum-low medium for 24 hours. The cell-free space was photographed by microscope at 0 h and 24 h. Transwell assay was carried out in 24-well plates, The upper chamber without Matrigel (for migration) or with Matrigel (for invasion), added to cell suspension (2 × 10^4^ cells/200 mL) without FBS. Subsequently, 800 μL medium with 10% FBS was supplemented into the lower chamber. After 24 hours of culture, the migrated or invasive cells were stained with crystal violet. Finally, the chamber was photographed under a microscope [27].

### Cell transfection

PYCR1 siRNA and NC siRNA (GenePharma Company, Shanghai, China) transfected cells using lipofectamine 2000 reagent (Invitrogen, Carlsbad, CA, USA). Firstly, the cells were grown to 50–60% confluence in 6-well plates before transfection. Secondly, the cells were transfected with PYCR1 siRNA and NC siRNA for 6 hours. Finally, after another 48 hours, western blotting and real-time PCR were performed to determine reduction efficiency. The sequence of the siRNAs used is as follows: NC, 5’-UUUUCCGAACGUGUCACGUTT −3’ (sense); siRNA2, 5’-GCCCACAAGAUAAUGGCUATT-3’(sense); siRNA3, 5’-GAAGAAGCUGUCAGCGUUUTT −3’ [28].

### RNA isolation and RT-qPCR

According to previous test methods [29], total RNA was extracted with TRIzol reagent(Invitrogen). Total RNA was subjected to first-strand cDNA synthesis by using a PrimeScript RT reagent kit (Takara, Shiga, Japan), and assessing gene expression was performed using SYBR-Green PCR Master Mix (Takara, Shiga, Japan). The results were analyzed with the 2− ^ΔΔCT^ method. The primer sequences were listed as follows: PYCR1: Forward: 5’-TCCATTGAGAAGAAGCTGTCAG −3’; Reverse: 5’-CATCAATCAGGTCCTCTTCCAC −3’; β-actin: Forward 5’-CCTGGCACCCAGCACAAT-3’, Reverse 5’-GGGCCGGAC TCGTCATAC-3’.

### Western blotting analysis

Western blot was used to detect protein expression level according to a previous study [30]. Total protein was extracted from cells lysed in RIPA lysis buffer (Beyotime, Shanghai, China). About 20–50 μg protein was separated via 10% SDS-PAGE and transferred to 0.45 µm PVDF membranes. After 5% nonfat milk blocking at room temperature for 2 h (phopho-proteins are blocked with 5% BSA solution), the membranes were probed with primary antibody at 4°C overnight, were incubated with an anti-rabbit secondary antibody (1:2000) (ProteinTech Group, Chicago, USA)for 2 h at room temperature. The signals were detected using an ECL kit (Thermo Fisher Scientific, Inc).

### Immunofluorescence

Cells seeded onto 35 mm petri dish with glass coverslips at the bottom for 48 h, fixed with 100% cold methanol for 20 min at room temperature, then permeabilized with Triton-X100 in PBS for 20 min. Cells were blocked for 2 h with 1% BSA and incubated with rabbit anti- LC3-I/II antibodies (1:500) overnight at 4°C, then incubated with goat anti-rabbit IgG AF488 (ProteinTech Group) at room temperature for 2 h in the dark. Eventually, the cells were co-stained with DAPI for 2 min. A laser scanning microscope acquired images to assess the fluorescence signal of LC3-I/II [31].

### Statistical analysis

SPSS 16.0, Photoshop, and GraphPad Prism 8 were used to analyze the experimental data. The independent sample t-test was applied to determine the difference between the control group and the treatment group. P < 0.05 was considered statistically significant. *, ** and *** indicated the p-values from t-tests are significant at 0.05, 0.01and0.001, respectively. At least three independent experiments confirmed all results.

## Results

### The anticancer effects of SK in HCC cells

**Figure 1.**
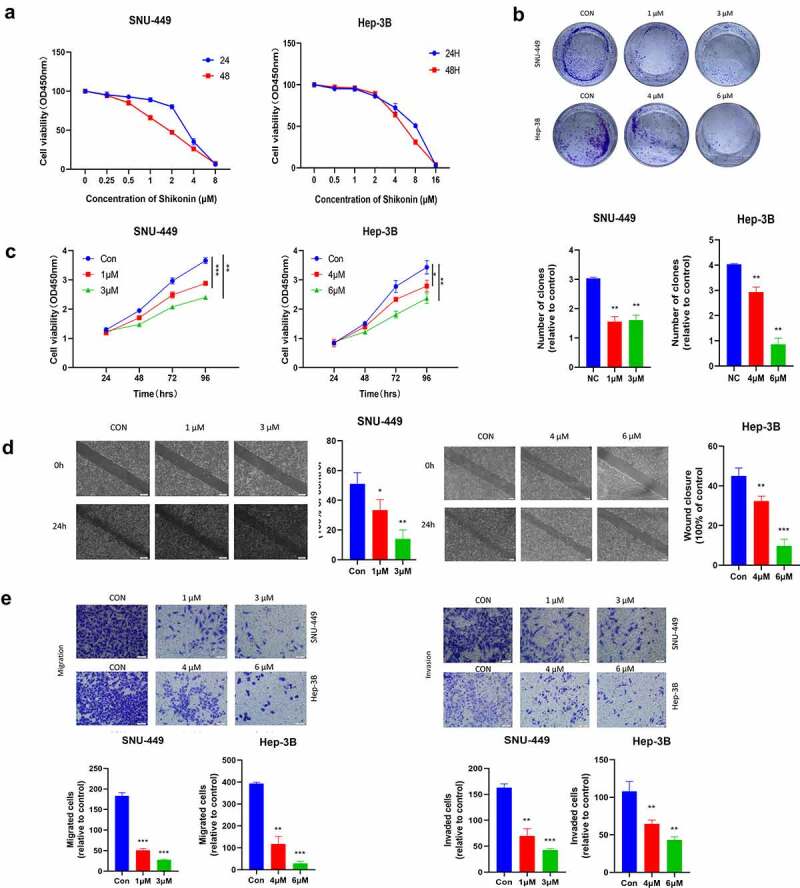
The Anticancer effects of SK in HCC cells. **a**.The cells were incubated with various concentrations of SK for 24 and 48 h, and cell viability was measured by CCK8 assay. **b**. After 3 or 5 μM SK treated SNU-449 and Hep-3B cells for 24 h, 48 h, 72, and 96 h, the CCK8 assay detected the cell proliferation. **c**. Colony formation assay was employed to evaluate the proliferation of HCC cells. The histogram represents the number of repeated bacteriolysis in each group based on colony formation measurements **d**. A wound-healing assay measured the motor ability of HCC cells. Histograms provide quantitative data on wound healing. **e, f**. The Transwell assay detected the migration and invasion of HCC cells. Compared with the control group, * P < 0.05, ** P < 0.01, *** P < 0.001.

To define the antitumoral role of SK in liver cancer cells, we detected the cytotoxicity of SNU-449 and Hep-3B cells with different doses of SK for 24 h and 48 h. The CCK-8 assay showed that SK exposure reduced HCC cell viability in a time- and dose-dependent manner (Figure 1a). Specifically, the IC50 value of SNU-449 cells at 48 hours was 2.03 ± 0.02 μM, but the inhibitory effect on Hep-3B cells was weaker than the SNU-449, the IC50 value at 48 h was 5.04 ± 0.26 μM, indicating that SNU-449 cells are more susceptible to SK than Hep-3B cells. In subsequent assays, we preferred 1 μM and 3 μM SK for treating SNU-449 cells, 4 μM and 6 μM SK for Hep-3B cells. SK governs cell growth and motility in tumor cells. The CCK8 assay showed that the inhibition effect of SK on proliferation rates increased with the increase of SK concentration (Figure 1c). The clone formation assay results consistently displayed that SK significantly attenuated the number of cloned cells (Figure 1b).

Moreover, carrying the transwell assays with or without coating by matrigel, compared to the control group, the number of SNU-449 and Hep-3B cells passing through the chamber in the SK group was much fewer (Figure 1e). The wound healing assay also demonstrated that different concentrations of SK aroused delay of wound closure in both SNU-449 and Hep-3B cells (Figure 1d). Altogether, the SK could contribute to the inhibition of viability, proliferation, and mobility of HCC cells and have the antineoplastic function.

### SK induced apoptosis and autophagy in HCC cells

**Figure 2.**
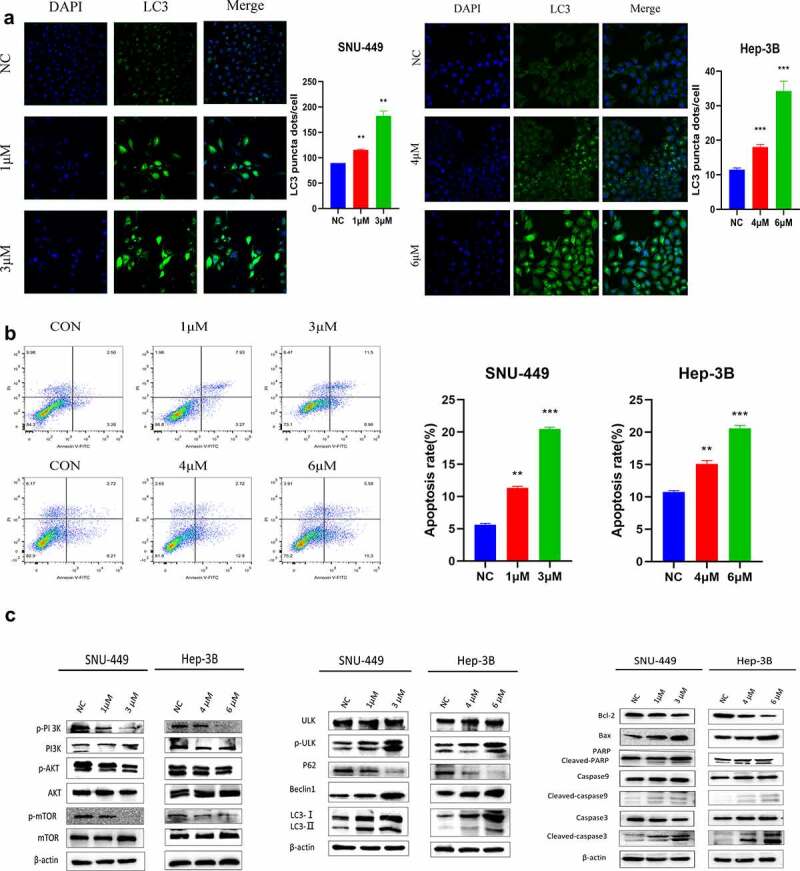
SK induces apoptosis and autophagy in HCC cells. **a**. Apoptosis was detected by annexin V/PI double staining. The results are expressed as a percentage of the control, which is set to 100%. **b**. The LC3 puncta were examined using confocal microscopy and were quantified. LC3 is shown in green. DAPI, shown in blue, stained the nuclei. Confocal microscope was taken at ×20. **c**. After being treated with different concentrations of SK for 48 hours, apoptosis (RAPA, caspase3, caspase9, Bax, Bcl2), autophagy (p-ULK, ULK, Bclin1, p62, LC3) and pathway (p-PI3K, PI3K, p-Akt, Akt, p-mTOR and mTOR related proteins expression was analyzed by Western blot. The data are expressed as the mean ± standard deviation of three independent experiments. * P < 0.05, ** P < 0.01, *** P < 0.001.

To evaluate whether autophagy and apoptosis are involved in the cytotoxicity of SK to HCC cells, we first studied some autophagy proteins during the full-length processing from autophagy marker LC3-I to LC3-II. We discovered that the LC3-I/II protein expression was raised in a dose-dependent manner in SK-treated(0, 1, 3 or 0, 4, 6 μM) HCC cells. Besides, the expression of p-ULK, Beclin-1autophagy protein was up-regulated p62 protein decreased (Figure 2c). To visualize LC3 accumulation, immunofluorescence analysis was carried out. Compared with the control group, LC3 green fluorescence spots in the SK treatment group (1, 3 or 4, 6 μM) increased, consistent with the Western blotting results (Figure 2a). It indicates that SK treatment triggers autophagy flux in HCC cells. In addition, we evaluated the apoptotic rate of SNU-449 and Hep-3B cells after distinct concentrations of SK treatment for 48 h. Annexin V-FITC/PI showed that SNU-449 and Hep-3B apoptosis cells gradually increased in SK groups contrasted with the NC group (Figure 2b). Western blot indicated decreased apoptosis protein levels of cleaved-caspase3, cleaved-caspase9, cleaved-PARP, and Bax/Bcl-2 in SNU-449 and Hep-3B cells. The results were consistent with flow cytometry, suggesting that SK may induce apoptosis of HCC cells (Figure 2c). The PI3K/Akt/mTOR signaling pathway enters into regulating multiple cellular biological activities, including in the cell cycle, proliferation, apoptosis, and autophagy, etc. To clarify the potential molecular mechanism of SK mediated effect, the expressions of PI3K, Akt, and mTOR protein was determined by Western blot assay using phosphorylated antibodies. The results showed that SK appreciably diminished p-PI3K, p-Akt, and p-mTOR protein levels, while the total protein level remained unchanged (Figure 2c). Based on the above research, we suppose that SK probably activates autophagy and apoptosis by inhibiting PI3K/Akt/mTOR signal.

### The anticancer effects of knockdown of PYCR1 in HCC cells

**Figure 3.**
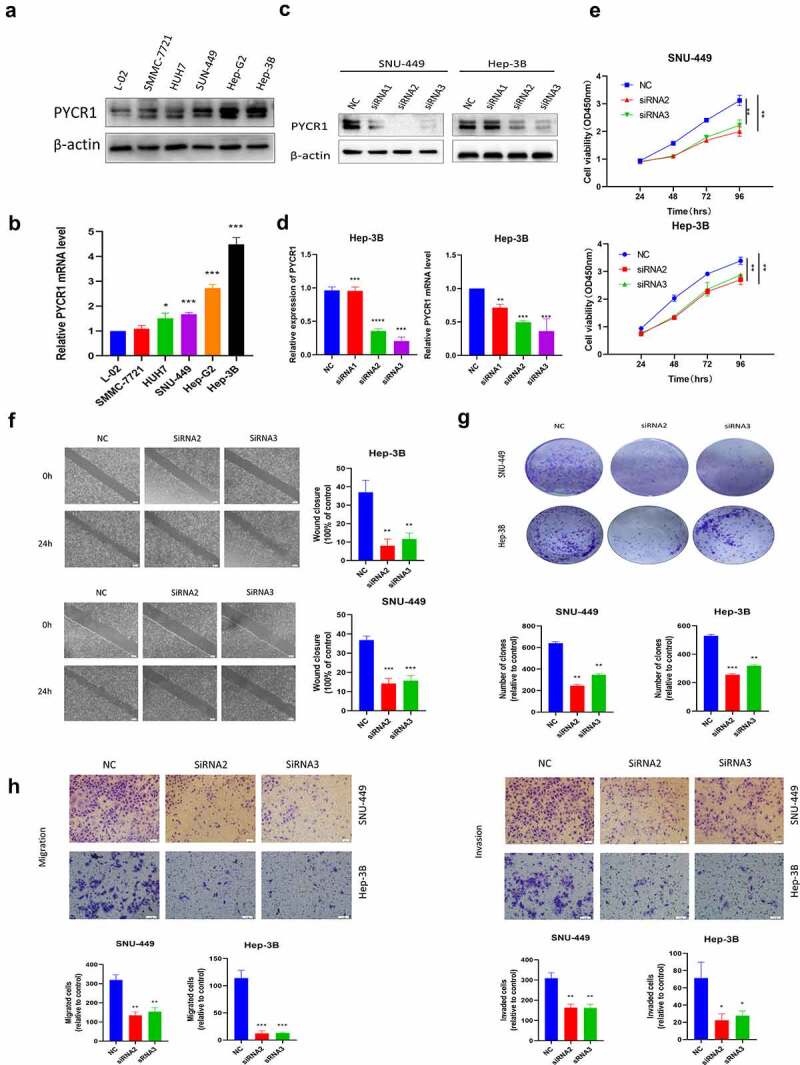
The Anticancer effects of Knockdown of PYCR1 in HCC cells. **a**. Relative levels of PYCR1 expression in LO2, SMMC-7721, Huh-7, Hep-3B, SNU-449, BEL-7404, and HepG2 were examined via RT-qPCR and Western blot. **b**. HCC cells were transfected with siRNA and the expression level of PYCR1. **c**. The CCK8 assay was employed to detect the viability of HCC cells. **d**. Colony formation assay was performed in SNU-449and Hep-3B cells treated with SK. **e**. Migration and invasion of HCC cells treated with SK for 24 hours by Transwell assay. **f**. The wound-healing assay obtained images at 0 and 24 hours after scratch. * P < 0.05; ** P < 0.01; *** P < 0.001; **** P < 0.0001.

To study the function of PYCR1 in tumorigenesis, we firstly identified the expression pattern of PYCR1 in liver normal cell line LO2 and HCC cell lines SMMC-7721, Huh-7, Hep-3B, SNU-449, BEL-7404, and HepG2. The expression of PYCR1 in various HCC cells was considerably higher in contrast to in LO2 cells (Figure 3a, b), suggesting that the overexpression of PYCR1 may be engaged in the progression of HCC. SNU-449 and Hep-3B cell lines that exhibit high expression of PYCR1, which were transfected with siRNAs against PYCR1. As opposed to the control group, the PYCR1 mRNA and protein levels were inhibited (Figure 3c, d). Subsequently, the growth curves detected by the CCK8 assays showed that the cell growth of siPYCR1 groups significantly slowed down (Figure 3e). The colony formation assay displayed similar effects of silencing PYCR1 as presented in Figure 3g. Lastly, we inspected the force of silencing PYCR1 on the metastatic activity of HCC. The chamber assay dissected that in SNU-449 cell lines, fewer cells were migrated and invaded in siPYCR1 groups than NC (Figure 3h). We obtained the same results in the Hep-3B cell line (Figure 3h). Similarly, the scratch healing assay observed that depleting PYCR1 enlarges the width of wound healing than that of the NC group in SNU-449 and Hep-3B cell lines (figure 3f). These results showed that down-regulated PYCR1 significantly inhibited tumor metastasis and the cellular activity of SNU-449 and Hep-3B cells.

### Downregulation of PYCR1 induced apoptosis and autophagy in HCC cells

**Figure 4.**
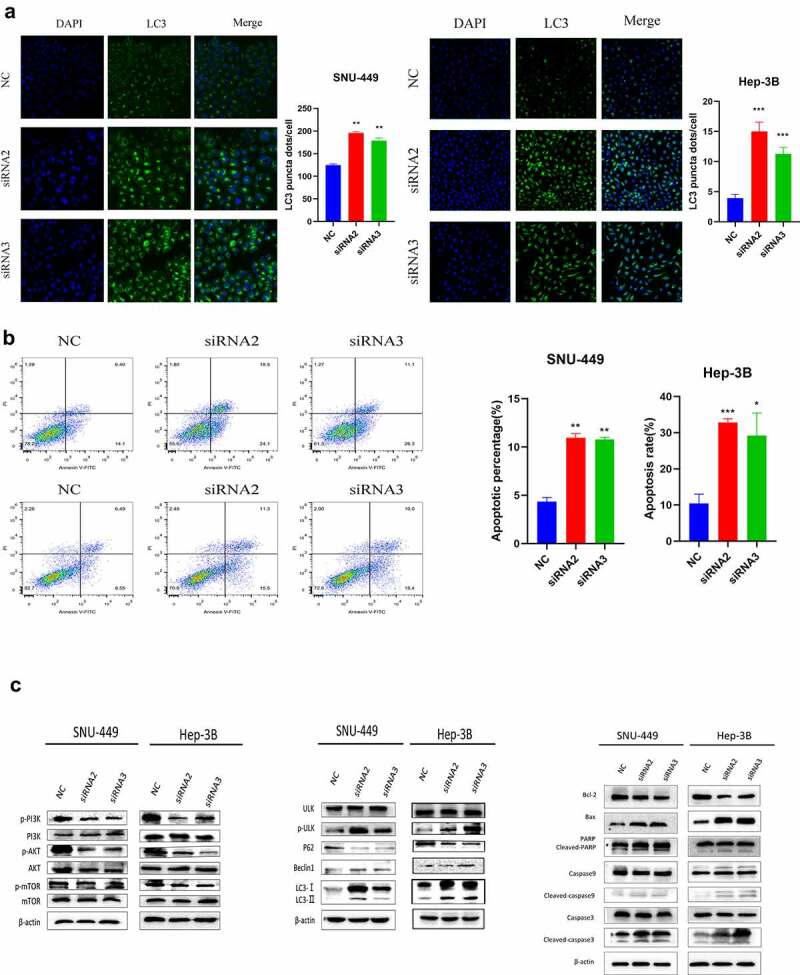
Downregulation of PYCR1 induces apoptosis and autophagy in HCC cells. **a**. SNU-449and Hep-3B cells expressing negative control or siPYCR1. LC3 signal was detected under a confocal microscope. The LC3 signal quantity of each cell was quantified. Scale bar: 100 μm. **b**. Apoptosis was detected by flow cytometry. **c**. Western blot analysis was used to determine the protein levels in both SNU-449and Hep-3B cells. * P < 0.05, ** P < 0.01, *** P < 0.001 vs the control(n = 3).

To appraise the mechanism of PYCR1 in autophagy and apoptosis of HCC cells. We found that siRNAs transfected SNU-449 and Hep-3B cells dramatically increased the accumulation of autophagic proteins p-ULK, Beclin-1, LC3-I/II, while p62 expression decreased with the NC group (Figure 4c). To further confirm this phenomenon, we detected the number of LC3 dots by immunofluorescence assay to evaluate the level of autophagysomes. Consistent with Immunoblotting results, we determined the silencing of PYCR1 resulted in the increase of LC3 fluorescence signal in HCC cells (Figure 4a). Simultaneously, flow cytometry analysis confirmed that the apoptosis rate of SNU-449 cells increased from 2.22% to 12.72% after siRNA treatment (Figure 4b). Similarly, the apoptosis rate of Hep-3B cells increased from 11.8% to 55.8% (Figure 4b). Consistently, apoptosis-related markers (cleaved-PARP, cleaved-caspase-3, cleaved-caspase-9, and Bax/Bcl-2) were up-regulated in HCC cells (Figure 4c). Moreover, to probe the force of siPYCR1 on PI3K/Akt/mTOR signaling pathway in HCC cells. Western blot data displayed that the phosphorylation levels of PI3K, Akt, and mTOR proteins in siPYCR1 groups were dramatically depressed against those in the NC group (Figure 4c). Summarily, the consequences implied that PYCR1 could foster autophagy and apoptosis by inhibiting PI3K/Akt/mTOR signaling.

### SK reinforces its anti-tumor effects by downregulating PYCR1 in HCC cells

**Figure 5.**
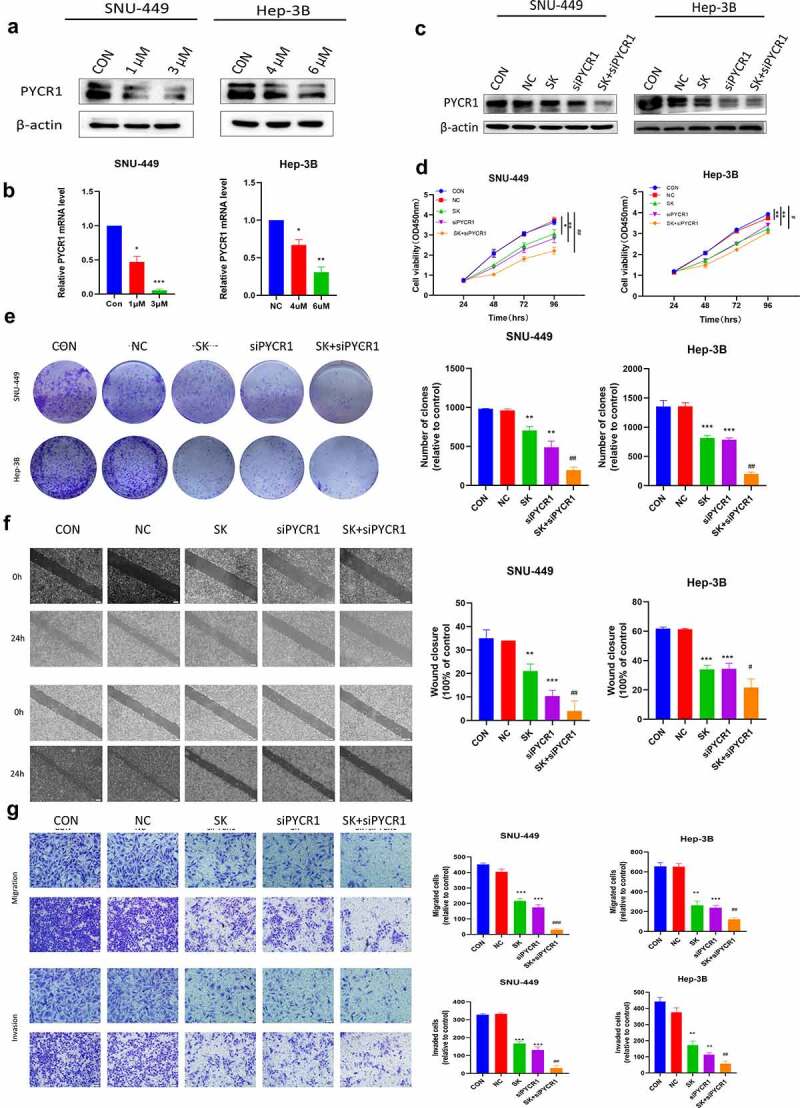
SK reinforces its anti-tumoral effects by downregulating PYCR1 in HCC Cells. **a, b, c**. Western blotting and RT-PCR detect protein and mRNA levels of PYCR1 in SNU-449and Hep-3B cells treated with SK (0, 1, and 3 μM) (0, 4, and 6 μM) for 48 h. **d**. The activity of each group in SNU-449and Hep-3B cells was detected by CCK8 assay. **e**. Colony formation assay was used to detect the growth of SNU-449and Hep-3B cells. **f**. Wound healing assays were performed on SNU-449and Hep-3B cells. The right panel displays a histogram of the results. Scale bar: 200 μm. **g**. Migration and invasion assay was carried out on SNU-449and Hep-3B cells. Scale bar: 50 μ m. *P < 0.05 for SK or siPYCR1vs the control; ##p < 0.01 for SK +siPYCR1 vs SK only.

PYCR1 functions as a critical oncoprotein in carcinogenesis. We explored whether SK performed an anti-tumoral effect via suppressing PYCR1 in live cancer cells. To attain this purpose, we utilized RT-PCR and Western blotting assay to appraise the expression of PYCR1 in SNU-449 and Hep-3B cells after SK treatments. Our data showed that SK significantly down-regulated the expression of PYCR1 at both mRNA and protein levels in SNU-449 and Hep-3B cells by the concentration-dependent fashion (Figure 5a, b). This discovery implied that PYCR1 restraint by SK possibly is a rationale for the SK-mediated repression of tumors. To investigate the anti-tumor activity of SK on HCC cells after interfering with PYCR1. We transfected HCC cells with siRNA and siNC to structure siPYCR1 and NC group, then, we respectively used 2 μM and 5 μM SK to treat SNU-449 and Hep-3B cells as SK group, and untreated liver cancer cells as controls (CON), 2 μM or 5 μM SK and siPYCR1 treatment cell together as SK+siPYCR1 group. Compared to the SK group or the siPYCR1group, our immunoblotting illustrated that the PYCR1 levels of the SK+siPYCR1 group were considerably lower (Figure 5c). The CCK8 data revealed that SK in amalgamation with PYCR1 siRNA transfection diminished cell viability to a lower level in contrast with single SK exposure or PYCR1 siRNA treatment only (Figure 5d). Similarly, the clone formation assay was employed to analyze the number of cloned cells after SK and siPYCR1 combination treatments. The results were consistent with the CCK8 assay. Cloned cells of the SK+siPYCR1 group were prominently reduced (Figure 5e). Furthermore, the transwell assays decreased migrated and invaded cells by cotreating the SNU-449 and Hep-3B cells with SK andPYCR1 siRNA transfection (Figure 5g). Finally, the wound healing assay showed that the SK and PYCR1 siRNA association aroused a tremendous lessening in live cancer cells’ wound healing ability than after a single treatment (figure 5f). Thus, these data demonstrate that SK may restrain the migration, invasion, and proliferation of HCC cells, and SK induces these processes by downregulation of PYCR1.

### SK induced apoptosis and autophagy of HCC cells by downregulated PYCR1 via inhibited PI3K/Akt/mTOR pathway

**Figure 6.**
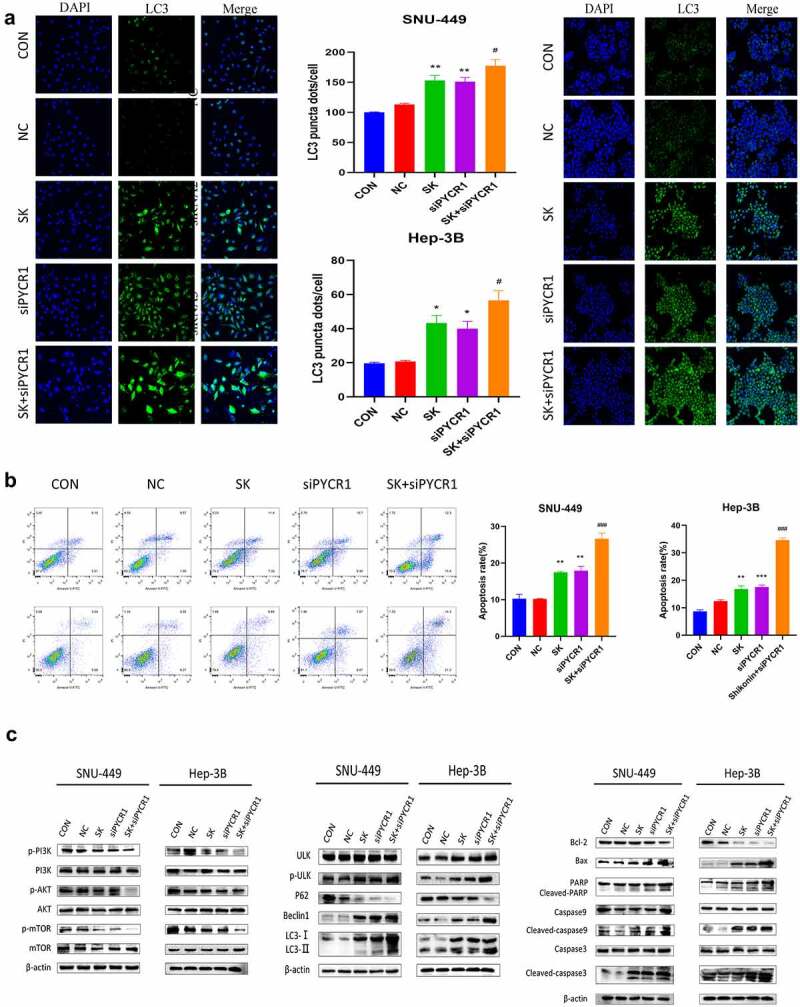
SK induced apoptosis and autophagy of HCC cells by downregulated PYCR1 via inhibited PI3K/Akt/mTOR pathway. **a**. Immunofluorescence assay (× 20) detected LC3 puncta of SNU-449and Hep-3B cells treated with SK and siPYCR1transfection for 24 h. **b**. The apoptosis of SNU-449and Hep-3B cells was detected by flow cytometry. **c**. The expression of pathway protein was detected by Western blotting, with β- Actin, was used as a loading control. Data are presented as the mean ± SD of three independent experiments (n = 3, * P < 0.05 for SK vs CON or NC, #P < 0.05, SK +siPYCR1 vs SK).

To evaluate whether SK downregulated PYCR1 to Induce apoptosis and autophagy via inhibiting the PI3K/Akt/mTOR pathway in HCC cells. Immunofluorescence assay data exhibited that the amount of LC3 fluorescent dots was remarkably increased in HCC cells with the combination treatment of PYCR1 siRNA and SK exposure for 48 h than an only single treatment (Figure 6a). In support of these data, Western blotting appeared that the protein levels of Beclin 1, p-ULK, and LC3-I/II raised most distinctly after PYCR1 interference and SK treated for 48 h. In contrast, P62 protein expression was dramatically decreased (Figure 6c). PYCR1 downregulation enhanced SK-triggered induction of cell autophagy compared to single treatment in SNU-449 and Hep-3B cells. In addition, our flow cytometry demonstrated that the SK or siPYCR1 group had the number of apoptotic cells was considerably lower as against the combination treatment. (Figure 6b). Similarly, Western blotting data showed that SK alone or PYCR1 suppression alone increased cleaved-caspase3, cleaved-caspase9, cleaved-PARP, and Bax/Bcl-2 levels compared to the control. Still, that union of both presented a more strengthened effect (Figure 6c). These data suggest that the downregulation of PYCR1 promotes SK-induced apoptosis and autophagy in HCC cells. Subsequently, we further illuminate the potential mechanism of SK Inhibited PYCR1 induced apoptosis and autophagy. Compared to the SK or siPYCR1 alone-treated group, Western blotting data showed that PI3K/AKT/mTOR signal pathway protein expression was significantly reduced after pretreated with the combination. There was a diminution in the expressions of phosphorylated PI3K, phosphorylated AKT, and phosphorylated mTOR, while the total protein remained unchanged (Figure 6c). In summation, siPYCR1 transfection increased the reduction of the pathway protein induced by SK treatment in SNU-449 and Hep-3B cells. It revealed that SK suppressed PYCR1 induced apoptosis and autophagy by restraining the PI3K/AKT/mTOR signaling pathways.

## Discussion

There is evidence that PYCR1 plays a pivotal function in the occurrence and advancement of human cancer, including liver [10,32]. One research group based on bioinformatics technology, reported that the PYCR1 was overexpressed in HCC cells. It played a multifaceted regulatory assignment in various biological pathways, such as cell communication, cell growth, cell migration, mitogen-activated protein kinase cascade, etc [33]. Another group observed that PYCR1 gene silencing could inhibit the proliferation of hepatoma cells, promote apoptosis, and significantly inhibit the volume and size of transplanted tumors in nude mice by regulating the JNK/IRS1 pathway [10]. At the same time, the PYCR1 gene silence also can constrain the invasion and migration of HCC cells, promote apoptosis and G1 arrest [14]. Because PYCR1 plays a cancer-promoting role in HCC, PYCR1 inactivation may become a molecular target for liver cancer therapeutic. In this research, we discovered that PYCR1 was raised to different degrees in HCC cells. We downregulated the PYCR1 gene expression of Hep-3B and SNU-449 cells by siRNA fragment. We found that PYCR1 gene knockdown inhibited the migration, invasion, and proliferation of HCC cells. In addition, we also found that siPYCR1 activated autophagy and apoptosis of HCC cells by suppressing PI3K/Akt/mTOR signaling pathway. PYCR1 may is a new prognostic marker and a potential therapeutic target for liver cancer.

SK is a small molecular naphthoquinone compound, which has been proved to possess many effects, such as anti-inflammatory, anti-virus, liver protection, anti-oxidation, anti-tumor, and immune regulation [34]. In animal models, high-dose administration of SK and its derivatives has been proven safe and well-tolerated [35]. So far, many studies have reported that SK can inhibit the occurrence and development of liver cancer. It can play an anti-tumor role by inhibiting pyruvate kinase M2 (PKM2), inhibiting the transcriptionally activated Nrf2 downstream target gene BAG3, and activating PKM2-AMPK-PGC1α signal pathway triggers mitochondrial dysfunction [36] [37,38]. Low-dose shikonin may inhibit the migration of hepatoma cells by downregulating the expression of vimentin and matrix metalloproteinase −2 and −9 [39]. It also improves the sensitivity of liver cancer cells to sorafenib and arsenic trioxide (ATO) treatment [36,40]. In addition, SK was also found to inhibit the growth of HCC cells in vitro and in vivo, which is related to inducing cell cycle arrest and promoting apoptosis [28,29]. However, the antineoplastic mechanism of SK in hepatoma cells is not precise. This study discovered that SK repressed the growth, migration, and invasion and triggered apoptosis of SNU-449 and Hep-3B cells. SK treatment increased the expression of LC3 and activated autophagy flux. At the same time, we also found that SK plays an anticancer role by inhibiting autophagy and apoptosis induced by PI3k/Akt mTOR pathway. These findings suggest that SK is an inducer of autophagy-dependent apoptosis and may be a promising clinical antitumor drug.

Previous studies have shown that SK inhibits the proliferation and induces apoptosis of hepatocellular carcinoma cells by inhibiting pyruvate kinase M2 (PKM2), while improving the sensitivity of Sola Feeney treatment [36]. Shikonin also inhibits breast cancer cell migration and invasion by up-regulating the expression of the tension protein homolog PTEN [41]. Given the above studies, we suspect that the antitumor effect of SK is related to PYCR1. Therefore, we detected the expression of PYCR1 after SK treatment by RT-PCR and Western blot. The findings demonstrate that SK reduced the expression of PYCR1 protein and mRNA in SNU-449 and Hep-3B cells to the greatest extent. To further confirm the vital force of PYCR1 in the antitumor process of SK, we detected the impact of SK on the cell proliferation, migration, and invasion after down-regulation of PYCR1. When the expression of PYCR1 was downregulated, it enhanced the inhibitory effect of SK on liver cancer cells. SK prominently represses cell proliferation, cell migration, and cell invasion compared with singly SK Treatment. These data indicate that PYCR1 holds a crucial role in SK’s anti-liver cancer. SK inhibits the growth of hepatoma cells by down-regulating PYCR1, highlight that SK in combined with PYCR1 inhibitor could be a promising therapeutic strategy for HCC therapy.

Autophagy is a self-regulatory mechanism in cells, which is related to cell death and survival [42]. At present, studies have found that there are changes in autophagy activity in various human tumors [43]. In tumor therapy, promoting autophagy activity and even autophagic death of tumor cells is a valuable new method to increase anti-tumor efficacy [44]. Studies have shown that SK can play an anti-tumor role in many cancers by activating autophagy and apoptosis, such as colorectal cancer, melanoma, and pancreatic cancer [23,24,45]. SK induces apoptosis and autophagy of colorectal cancer cells by targeting galectin-1 and JNK signaling pathways in vitro and in vivo. Galectin-1 is the target of SK for proteomic analysis [23]. In addition, SK can also inhibit autophagy to alleviate hepatic fibrosis by the platelet-activating factor-mitogen-activated protein kinase or transforming growth factor-beta1/Smad pathway [46,47]. However, SK and PYCR1 on autophagy and apoptosis of liver cancer cells are unclear.

In the current study, we observed that SK activated apoptosis and autophagy in a dose-dependent manner. At the same time, the combination treatment of SK and PYCR1 downregulation can significantly enhance SK-induced autophagy and apoptosis in HCC cells, inducing autophagy formation, LC3 redistribution, apoptotic rates, the levels of autophagy and apoptosis-related proteins. These findings indicated that autophagy might have a protective function in maintaining cell survival and proliferation under SK stress, andPYCR1 downregulation enhances this effect. This evidence indicates that the combination of SK and siPYCR1 probably become a potential strategy to improve the anti-tumor effect of SK, it provides a potential new therapeutic target for liver cancer.

Previous studies have demonstrated that autophagy is a multi-step regulatory process, and signal transduction is very complex, which humans have not fully mastered at present. PI3K/Akt/mTOR pathway is an essential intracellular signal pathway regulating cell survival, proliferation, autophagy, and growth [43,48]. Inactivated PI3K/Akt/mTOR signaling pathway can inhibit the survival of HCC cells and induce autophagy and apoptosis [49,50]. Drugs targeting the PI3K/Akt/mTOR signaling pathway may repress the tumor cell’s survival pathway and activate autophagy and apoptosis in cancer cells [49]. In addition, previous studies have shown that SK can induce apoptosis and autophagy in melanoma cells by activating the ROS pathway [24]. It also activates the autophagy of pancreatic cancer cells through PI3K/AKT signaling pathway [45]. It can also activate PI3K/ AKT signaling pathway inhibits apoptosis and autophagy to reduce hepatic ischemia/reperfusion injury [51]. In this study, with the decreased expression of phosphorylated PI3k, Akt and mTOR, the PI3K/Akt/mTOR pathway could be inhibited by SK in HCC cells. Furthermore, siPYCR1 downregulated phosphorylated PI3K, Akt, and mTOR can reinforce HCC cells’ sensitivity to SK. These results suggest that SK downregulated PYCR1, inhibiting PI3K/Akt/mTOR signaling pathway, inducing apoptosis and autophagy of HCC cells. The results of this study show that SK may be the anticancer treatment of liver cancer and the potential diagnostic marker of liver cancer

## Conclusion

In summary, we confirmed that SK and siPYCR1 could repress cell proliferation, migration, and invasion, induce apoptosis and autophagy. Moreover, our research indicates first-ever that evidence supporting the antitumor mechanism of SK may be related toPYCR1 in HCC cells. We believe that SK inhibits the activation of the PI3K/Akt/mTOR pathway by downregulating the expression of PYCR1 to induce autophagy and apoptosis of HCC cells. All these discoveries deliver a new perspective for us to understand the antitumor activity of SK.

## Supplementary Material

Supplemental Material
