# Supplementary figures and images for: Shikonin induces apoptosis and autophagy via downregulation of pyrroline-5-carboxylate reductase1 in hepatocellular carcinoma cells
Source: Bioengineered. 2022 Mar 16;13(3):7904–18. doi: 10.1080/21655979.2022.2052673 (PMC9208523; doi:10.1080/21655979.2022.2052673)

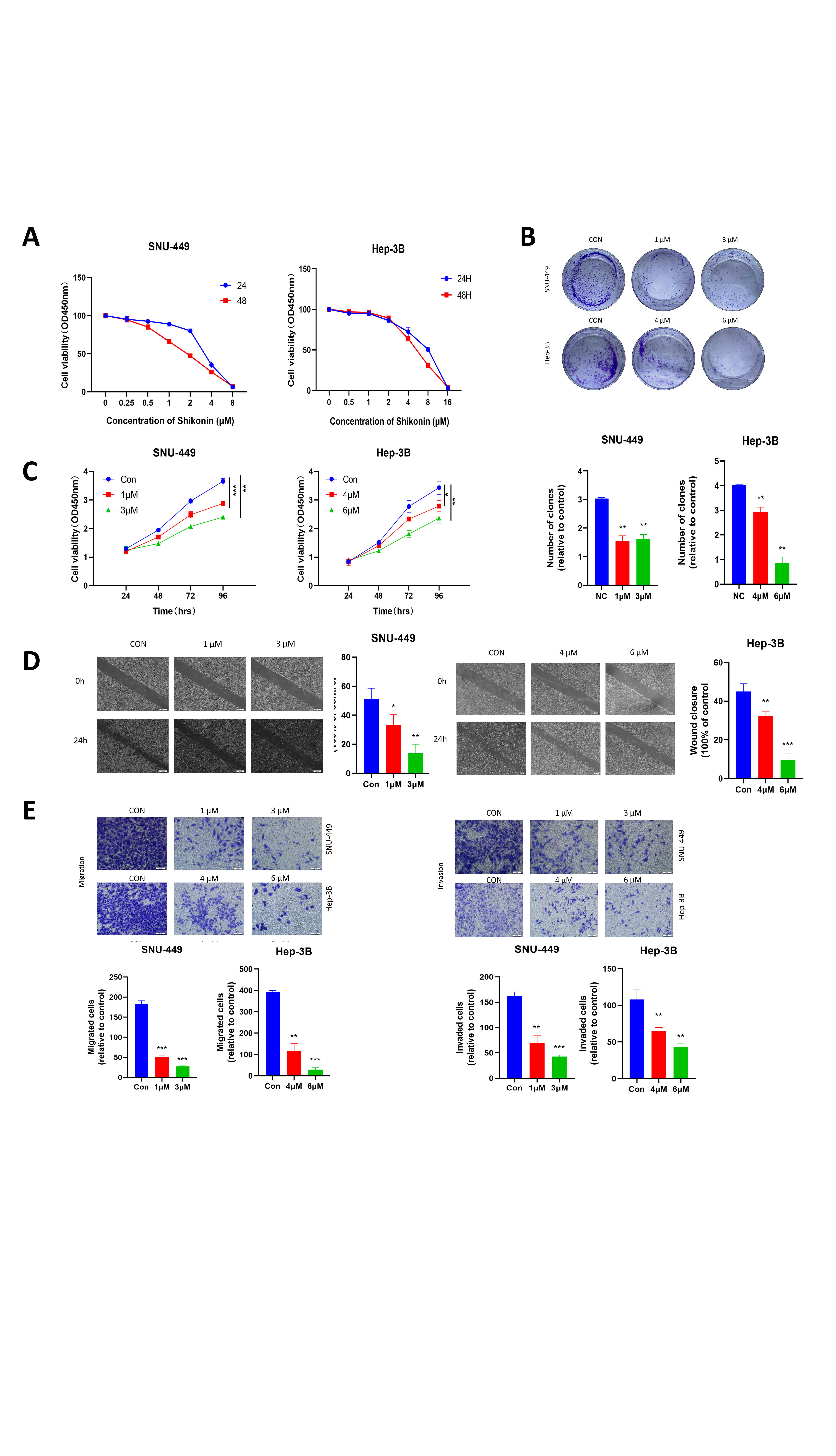

Supplement: Supplemental Material [file KBIE_A_2052673_SM2705.zip › 1.tif]

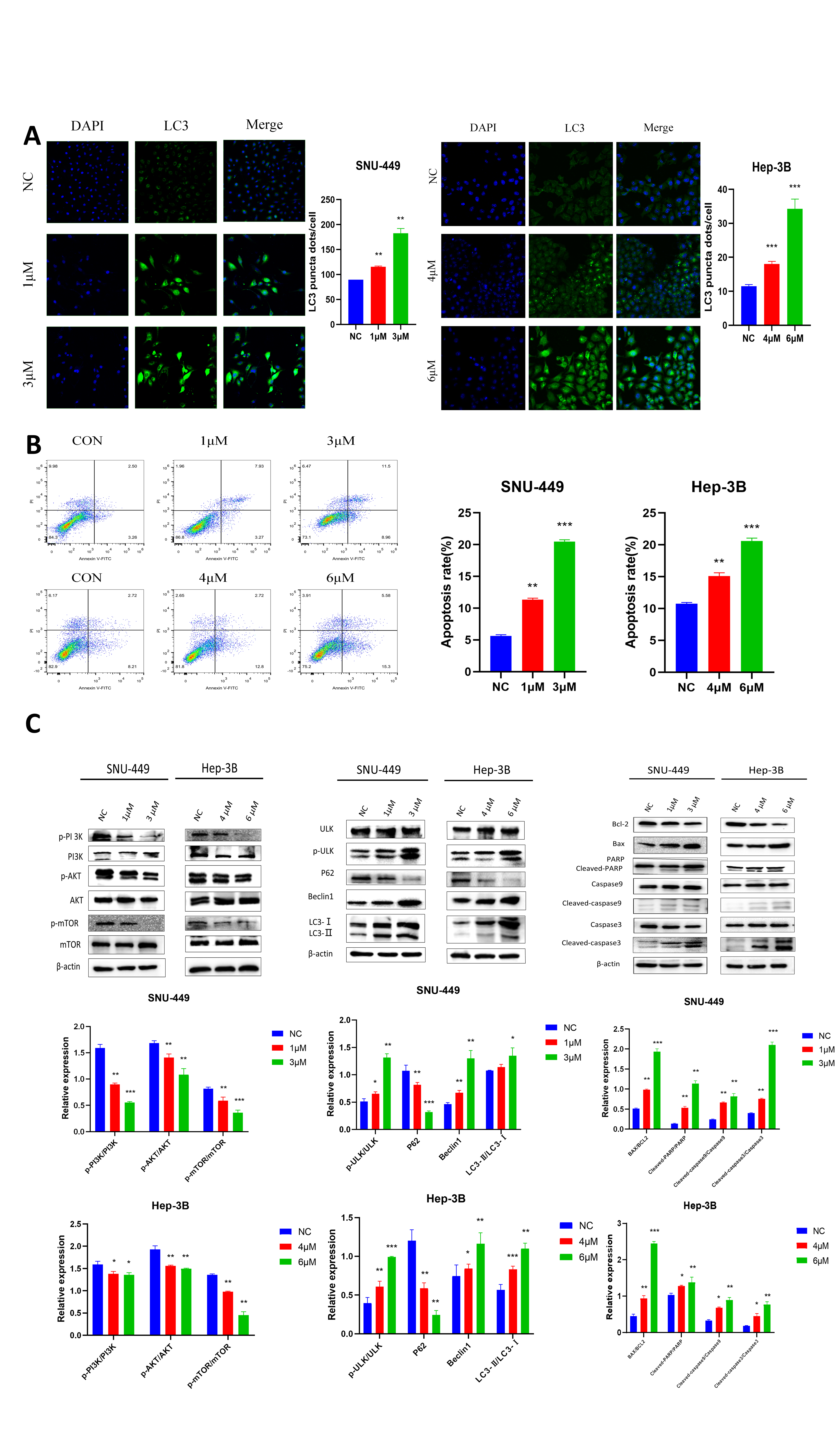

Supplement: Supplemental Material [file KBIE_A_2052673_SM2705.zip › 2.tif]

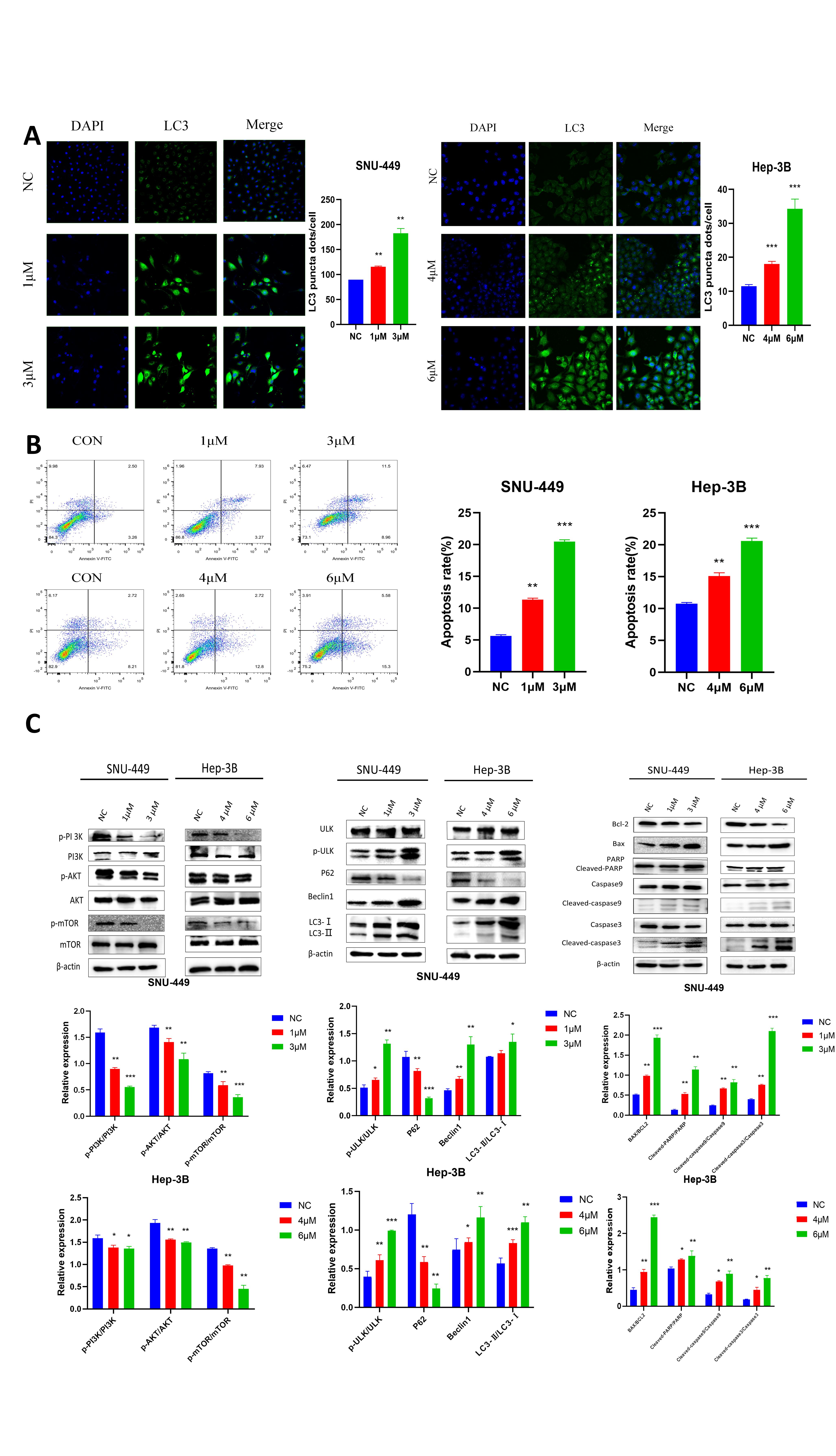

Supplement: Supplemental Material [file KBIE_A_2052673_SM2705.zip › 2_1.TIF]

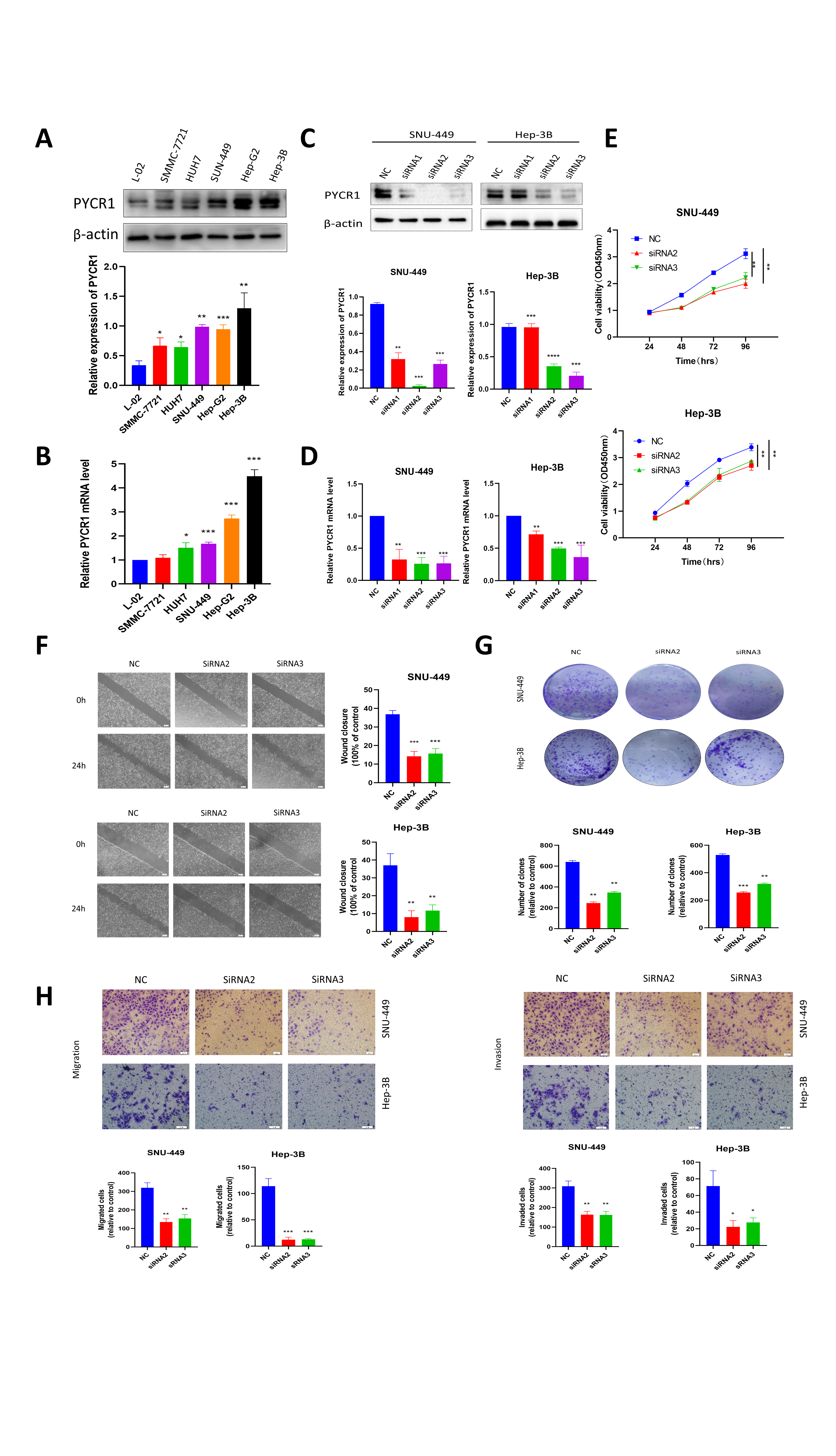

Supplement: Supplemental Material [file KBIE_A_2052673_SM2705.zip › 3.tif]

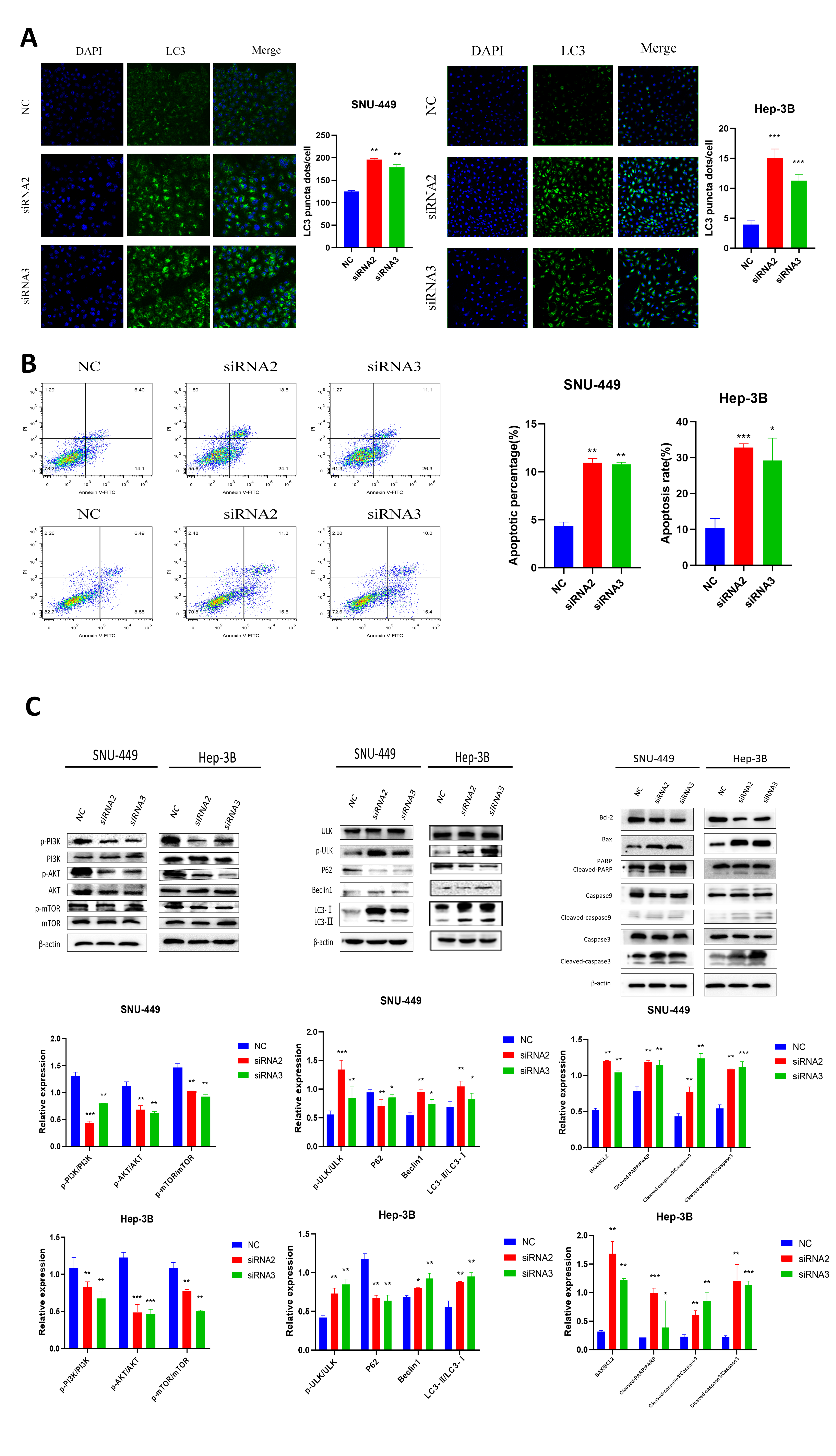

Supplement: Supplemental Material [file KBIE_A_2052673_SM2705.zip › 4.TIF]

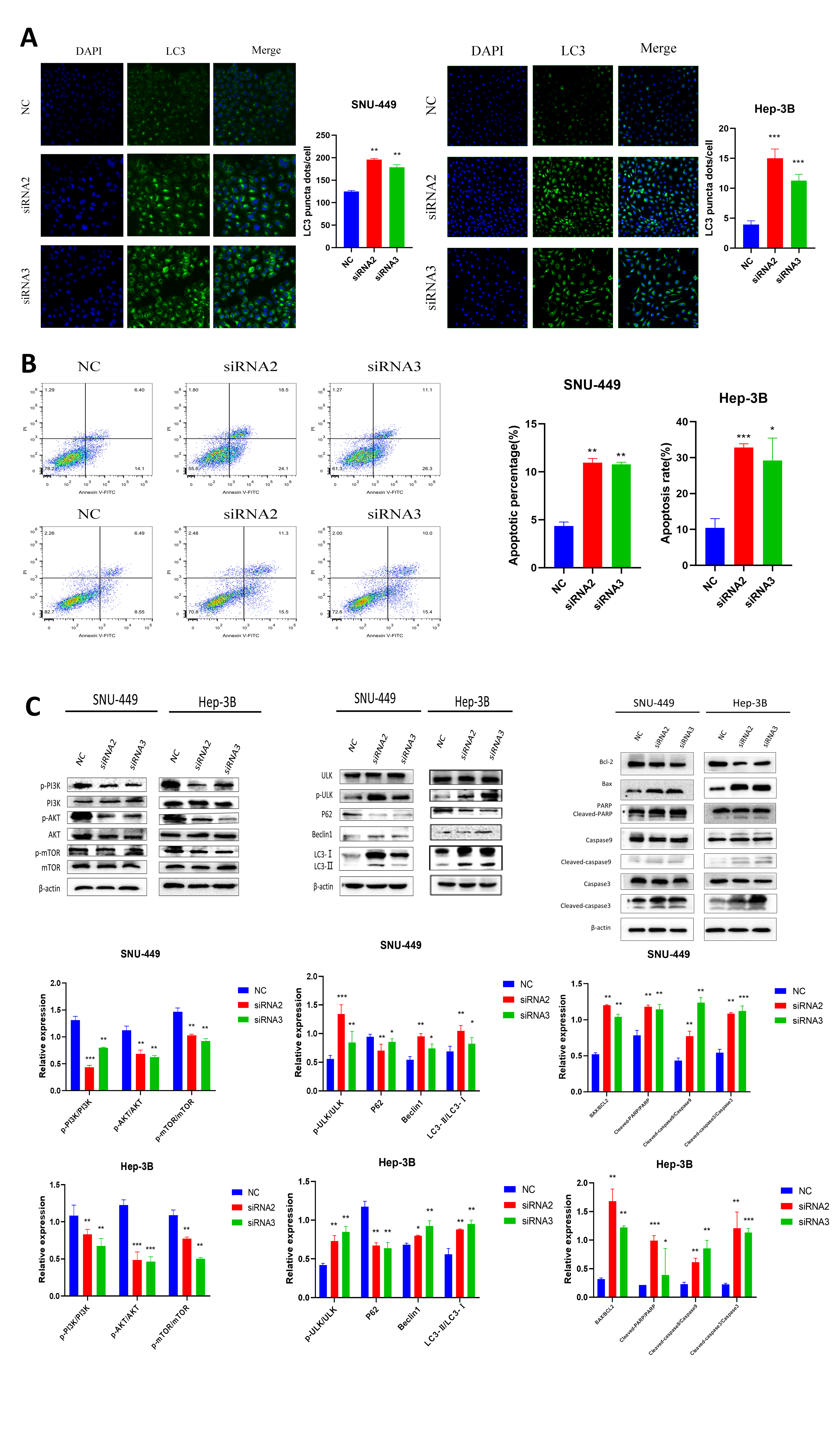

Supplement: Supplemental Material [file KBIE_A_2052673_SM2705.zip › 4_1.tif]

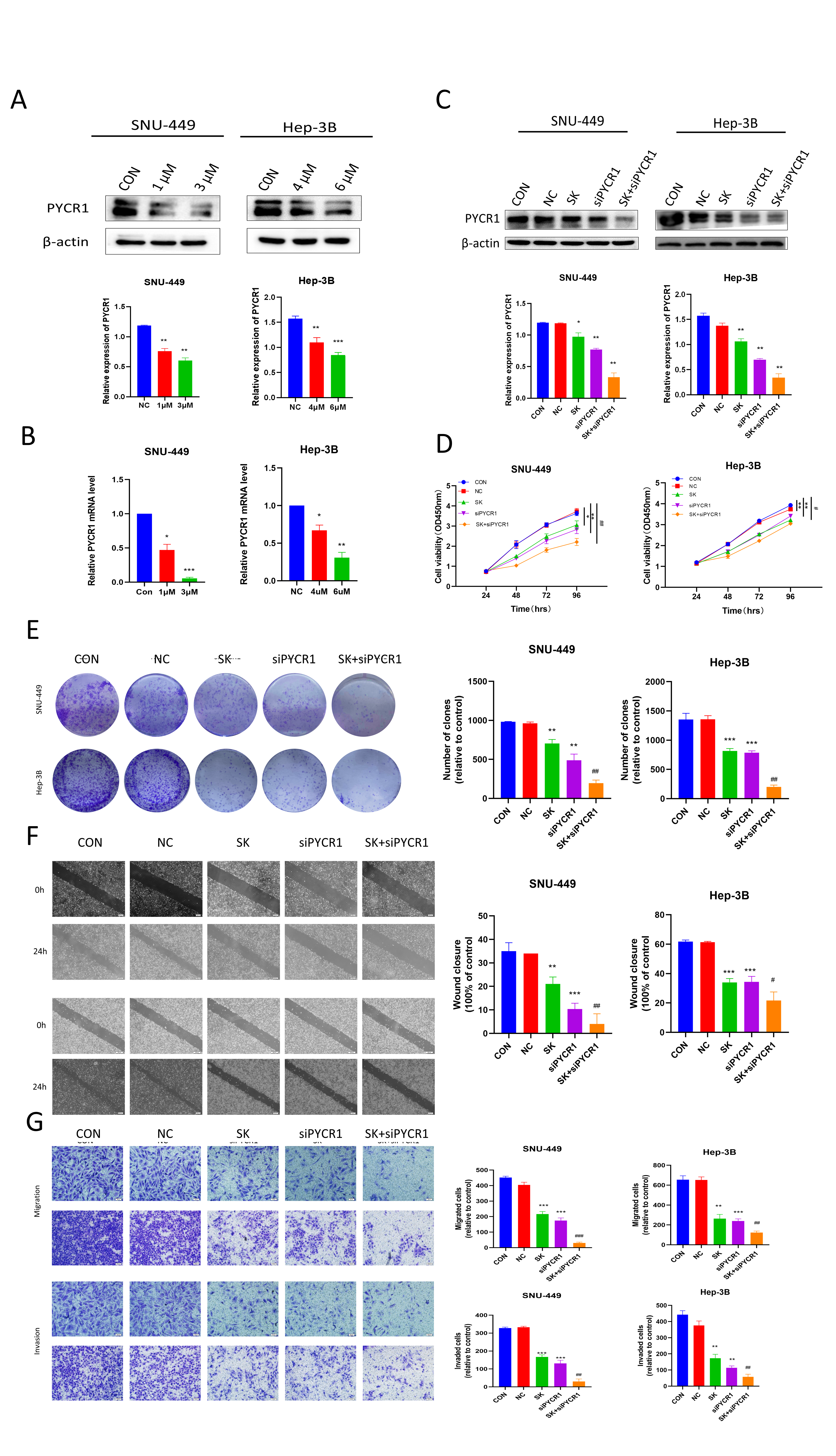

Supplement: Supplemental Material [file KBIE_A_2052673_SM2705.zip › 5.tif]

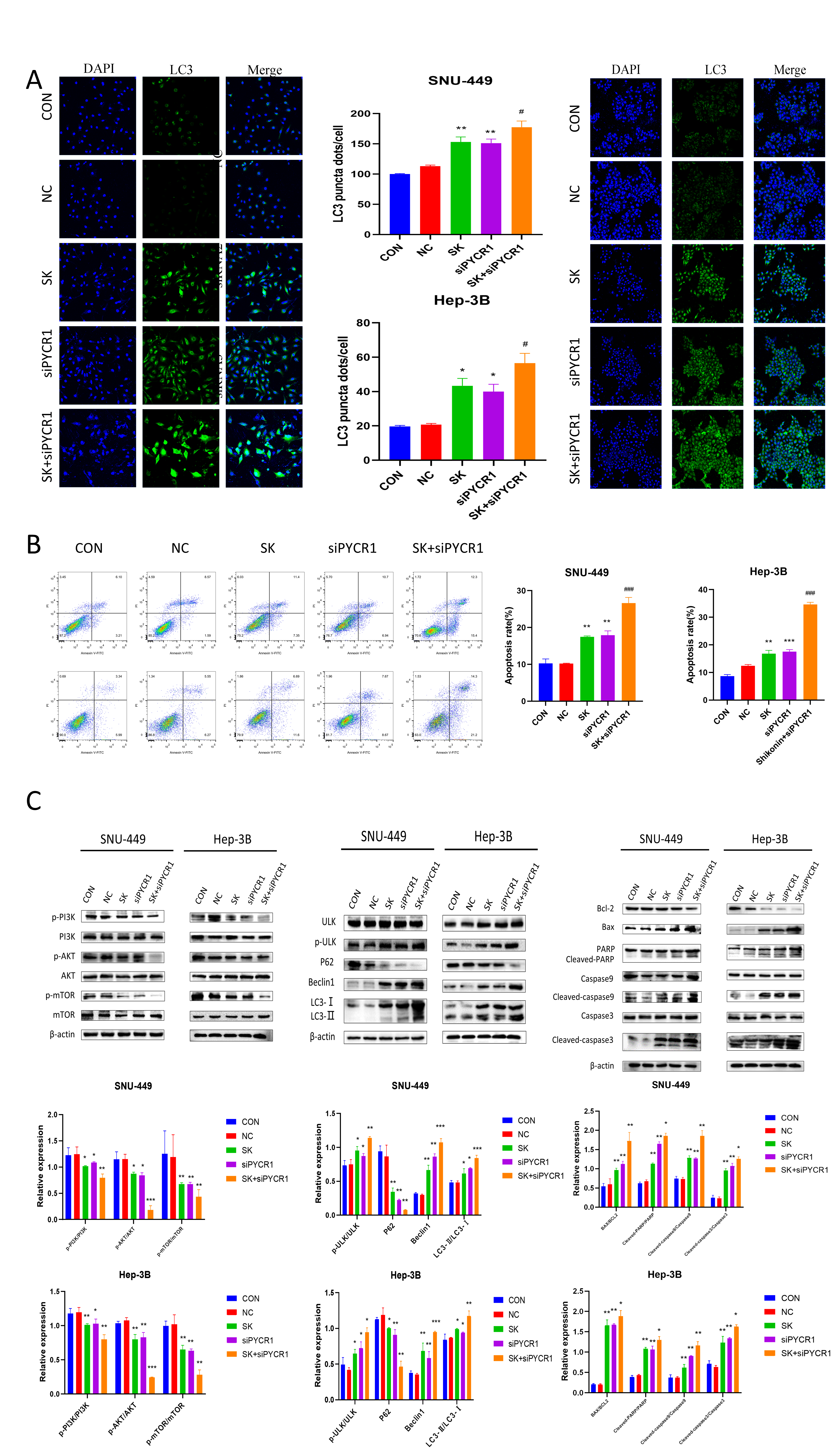

Supplement: Supplemental Material [file KBIE_A_2052673_SM2705.zip › 6.TIF]
